# Social Influence in Televised Election Debates: A Potential Distortion of Democracy

**DOI:** 10.1371/journal.pone.0018154

**Published:** 2011-03-30

**Authors:** Colin J. Davis, Jeffrey S. Bowers, Amina Memon

**Affiliations:** 1 Department of Psychology, Royal Holloway University of London, London, United Kingdom; 2 Department of Experimental Psychology, University of Bristol, Bristol, United Kingdom; Indiana University, United States of America

## Abstract

A recent innovation in televised election debates is a continuous response measure (commonly referred to as the “worm”) that allows viewers to track the response of a sample of undecided voters in real-time. A potential danger of presenting such data is that it may prevent people from making independent evaluations. We report an experiment with 150 participants in which we manipulated the worm and superimposed it on a live broadcast of a UK election debate. The majority of viewers were unaware that the worm had been manipulated, and yet we were able to influence their perception of who won the debate, their choice of preferred prime minister, and their voting intentions. We argue that there is an urgent need to reconsider the simultaneous broadcast of average response data with televised election debates.

## Introduction

Televised election debates were introduced in the United States in 1960, and now play a prominent role in the election campaigns of many countries, including Australia, Canada, France, Germany, Italy, Japan, the Netherlands, New Zealand, and the United Kingdom. In 2009, Afghanistan, Mongolia and Iran screened their first ever televised debates [1]. Such televised debates can trigger substantial shifts in voting intentions, and “winning” a debate has a significant positive impact on electoral support for the candidate's party, particularly among undecided voters, though such effects may be relatively short-lived [2]–[9]. In a close electoral race, winning a debate shortly before Election Day could determine the outcome of the election.

**Figure 1:**
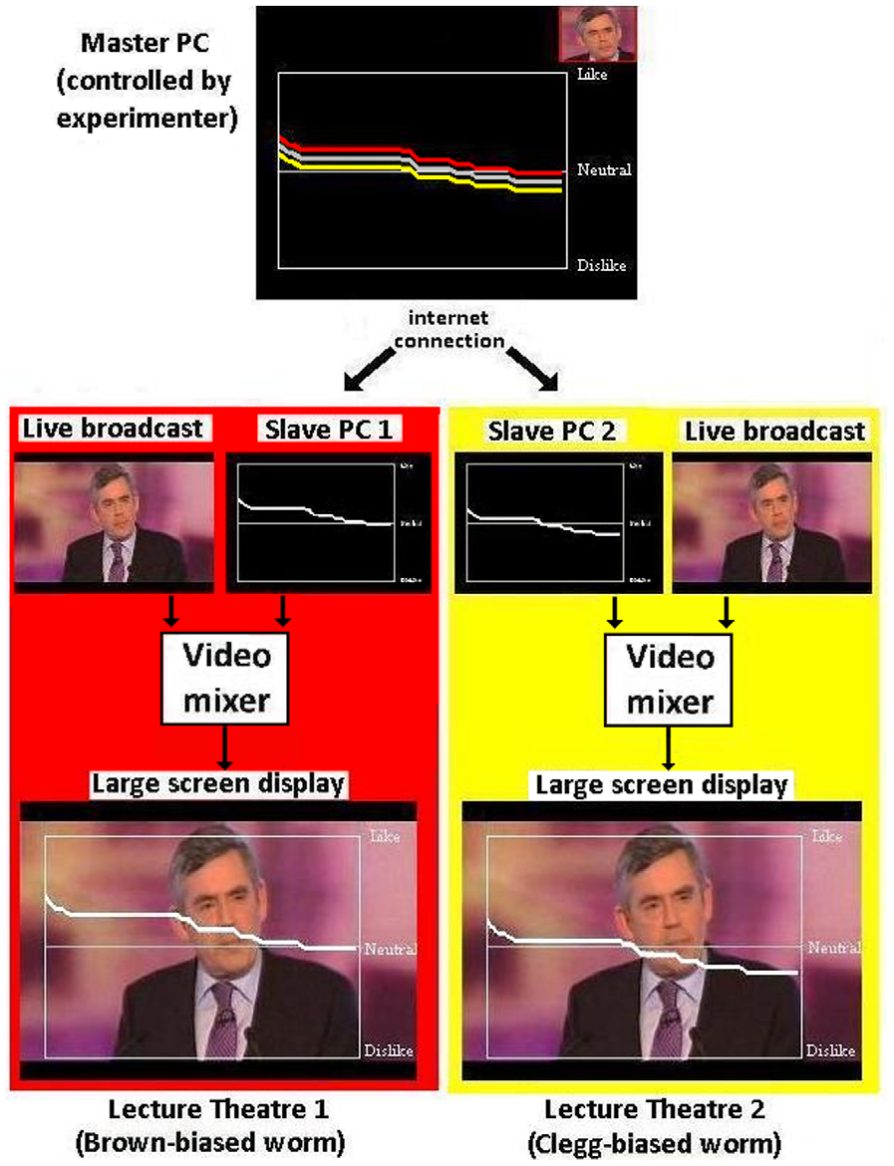
Examples of stimuli, and explanation of their production. (a) A screen shot from the first UK election debate (April 15, 2010), including worm, as shown on ITV.com. (b) Components involved in manipulation of worm and superposition on debate broadcast.

A feature of some recent televised election debates has been a real-time response measure – commonly referred to as “the worm” – which represents the average response of a small sample of undecided voters who watch the debate and use a handset to record their satisfaction with what the leaders are saying (e.g., the viewers turn a dial to the right to indicate approval, and to the left to indicate disapproval). These ratings are averaged over the entire sample in real-time and the average response is plotted using a moving line superimposed over the video of the debate (see Figure 1). The sample size is typically around twenty to thirty viewers; for example, CNN used a sample size of 30 voters to generate a worm in the 2008 US Presidential debates [10]. The worm graph allows viewers to see instantaneous reactions to the performance of the candidates, adding drama and interest to the debates. This technology has recently been adopted in elections in the US, UK, New Zealand, Australia, and given recent trends, is likely to be adopted more widely in the future. Experience in Australia and the United States has been that, given the choice, viewers prefer to watch election debate coverage that includes a worm, though the presentation of the worm has been the subject of controversy [10], [11].

Our question was whether the worm exerts a social influence effect. The psychological literature includes many demonstrations of how individuals can be strongly influenced by the opinions of others, especially (though not only) in situations involving uncertainty [12]–[15]. Social influence effects are partly normative, whereby observers strive to conform with their social group. However, social influence effects are observed even when normative factors are minimised (e.g., by making observers' responses anonymous and reducing the observer's identification with the group). People are motivated to be accurate, and when judgments or decisions are difficult, this motivation may prompt us to take information from others as evidence about reality [14]. Such informational influence effects have been reported in experiments in which observers make simple perceptual judgments [12]–[14], as well as those in which they respond to more complex real-world stimuli; for example, observers rate a joke as funnier (and are themselves more likely to laugh) if the joke is followed by laughter [16]. Social influence also affects people's memories [17], as illustrated by the memory conformity effect, in which eyewitnesses influence each other's memories for an event [18].

Two previous studies have investigated the influence of a worm-type graphic on viewer evaluations [19], [20; we thank an anonymous reviewer for bringing these studies to our attention]. In one study [19], participants watched video clips taken from the German version of the television program *Pop Idol* (*American Idol* in the US). They were told that the videos had been viewed previously by an audience of young adults, and that the responses of these adults were measured in real-time, and would be displayed as a graphic over the video. All participants watched the same clips, but there were three different versions of the worm graph, one that was positive, one that was negative, and one that fluctuated in a restricted range around a neutral baseline. Participants exposed to the negative version rated the performance more negatively than participants exposed to the neutral or positive versions; they also enjoyed the performance less. Interestingly, the ratings of participants exposed to the positive version did not differ from those of the participants exposed to the neutral version. Thus, exposure to the worm did have a clear social influence effect, but only in one direction: social influence was able to diminish performance evaluations but not improve them.

The extent to which social influence effects that have been observed in laboratory tasks might affect judgments of a real-world political debate is not clear. The participants in an election debate are themselves actively seeking to influence viewers, providing a rather different context from that in which social influence effects have previously been studied. Furthermore, social influence effects are weaker when the object of evaluation is more consequential [21]. Thus, a viewer of an election debate may be less susceptible to social influence effects, assuming that he or she has some stake in the outcome of the election. Another factor that might be expected to diminish social influence effects in this context is the fact that political attitudes are difficult to change. This tenacity is partly due to party-political affiliations, but also reflects the robustness of those policy attitudes that are important to an individual [22]. When a candidate in a political debate offers a policy position that differs from the viewers, the discrepancy is likely to elicit a more elaborative consideration than statements that are consistent with the viewer's own attitudes, and such elaborative processes are less vulnerable to social influence effects [23]. Thus, an apparently positive response from other viewers is unlikely to counteract the tendency for a viewer to negatively evaluate candidates whose statements disagree with her own attitudes. Each of the above-mentioned factors may cause us to doubt whether a televised worm graph has the potential to influence viewers' perceptions of an election debate.

There is one previous study that has tested the influence of the worm graph on viewers of election debates [20]. Participants, tested in groups of 15–20, watched short segments of the second Reagan-Mondale debate from the 1984 presidential election. Each student was given a handheld dial and shown how to use it to indicate their opinions during the debate. They were told that the line graph that they saw superimposed over the debate videotape showed the group's average opinion. In fact, the graph had been programmed in advance to favour either Reagan or Mondale. Participants who saw the pro-Reagan feedback rated Reagan's performance as better than Mondale's, whereas participants who saw the pro-Mondale feedback rated Mondale's performance as better than Reagan's. This finding suggests that the worm can have a powerful social influence effect on viewers of election debates. However, the ecological validity of this study can be criticised on a number of grounds. First, the excerpts that participants watched were from a debate that had occurred many years earlier. This could be problematic for various reasons: the issues discussed in the debate were not topical; it is likely that one of the candidates was not well-known to the viewers; the outcome of the election was (presumably) known to the viewers; and, perhaps most importantly, the participants were not able to vote for these candidates, and thus had no particular stake in the outcome of the debate. Second, in contrast to the usual experience of viewing debates, the participants in this study were required to make continuous ratings of their own opinion. This aspect of the study may have exaggerated social influence effects, especially if participants actively compared how the movements of the worm compared with their own movements of the dial. Furthermore, the participants' attention was explicitly drawn to the worm prior to the debate, e.g., they were shown how it could reflect their own movement of the dial, and they were given (misleading) details about the computation of the data plotted by the worm. Finally, participants in this study watched only excerpts of the debate, rather than the full 90-minute debate. These issues do not undermine the conclusions of this study (though see Ref. 10 for an alternative view), but they do raise the question of how likely its findings are to generalise to a more typical experience of viewing an election debate. Our study is better placed to address this question, as it involved participants watching an entire live election debate (for an election in which they were eligible to vote), without being required to make their own continuous ratings.

To test whether viewers of worm graphs are vulnerable to social influences, we asked two groups of 75 adults (students at Royal Holloway, University of London) to watch a live broadcast of the third (and final) 2010 UK election debate that included a worm of a similar format as in broadcasts of prior debates (see Figure 1). Unbeknownst to the participants, the worms seen by the two groups were manipulated by us to favour different candidates. In one group, the worm systematically favoured the incumbent, Gordon Brown, over the other two candidates. In the other group, the worm favoured Nick Clegg, the leader of the Liberal Democrat party. The worms seen by these two groups deviated by a fixed amount, in opposite directions, from a single baseline worm that was controlled by the experimenter. Our worms were superimposed on the live broadcast using video mixers (Figure 1). Our hypothesis was that perceptions of the debate would differ between the two groups in accord with the worm's bias.

## Methods

### Ethics Statement

The study was approved by the Ethics Committee of the Psychology Department at Royal Holloway, University of London. All participants provided written informed consent, and were fully debriefed at the conclusion of the experiment.

### Participants

The participants were 85 female and 65 male students at Royal Holloway, University of London; the majority (79%) were undergraduates, were of British nationality (77%), and were aged between 18 and 25 (95%). Participants received £20 for taking part. They were randomly assigned to one of two groups: Group 1 viewed a worm which favoured the incumbent Prime Minister Gordon Brown; Group 2 viewed a worm that favoured the Liberal Democrats leader Nick Clegg.

### Worm manipulation

The worm was controlled by an experimenter (CD) from his office in the Psychology Department. This control was achieved by means of two bespoke C++ programs. The first program (the “master”) ran on the experimenter's PC, and transmitted data, via an internet connection, to PCs in the two lecture theatres where the debate was watched. The latter PCs ran the second program (the “client”), which received the data and plotted a worm graph. This graph consisted of a white time series line framed by a white border on a black background. The minimum and maximum value of the y-axis were given the labels “Negative” and “Positive”, respectively, and there was also a horizontal axis with zero-intercept labelled “Neutral” (see Figure 1). The master program plotted the movements of three worms: the Brown-biased worm, the Clegg-biased worm and an intermediate worm. The experimenter pressed keys to move the intermediate worm up or down at one of three possible gradients; in the absence of input the worm traversed a line with a gradient of zero. The experimenter also used the program interface to indicate which candidate was currently speaking. This information was used by the program to compute the movements of the biased worms. When the current speaker corresponded to the worm's bias, movements in the positive direction were 25% steeper than the unbiased worm, and movements in the negative direction were 25% less steep. Conversely, when the current speaker corresponded to a candidate not favoured by a worm, movements in the positive direction were 25% less steep than the unbiased worm, and movements in the negative direction were 25% steeper. These speaker-dependent gradients meant that the two biased worms diverged (from each other and from the intermediate worm) fairly soon after Brown or Cameron began speaking, and rapidly approached the maximum distance from the intermediate worm. This maximum distance was 0.10 at the beginning of the debate, and increased to 0.15 after approximately 20 minutes. That is, when Brown was speaking, the Brown-biased worm was higher than the unbiased worm and the Clegg-biased worm was lower than the unbiased worm. When Cameron was speaking, the worms in both groups were lower than the unbiased worm. The first author adjusted the unbiased worm in response to the candidates in real time so that that the movements of the worms appeared plausible and responsive to the ongoing debate.

### Apparatus and technical details

Each lecture theatre was equipped with a desktop PC, a laptop computer, a vision mixer (Panasonic MX50) and a DVD recorder. The live broadcast from the BBC website provided one input to the mixer, while the desktop PC (which output the worm graphic) provided a separate input; the two inputs were combined using a luminance key-in technique that overlaid the worm on the live debate feed. The resulting signal was displayed on a large screen and also recorded in DVD format. Some example clips can be found here: http://www.pc.rhul.ac.uk/staff/c.davis/ElectionDebate/.

### Procedure

The study took place on the evening of the final of three UK election debates (29th April, 2010). Upon arrival, participants were directed to one of two different lecture theatres (depending on which group they had been randomly assigned to) and asked to complete the pre-debate questionnaire. The lead experimenter in each room then welcomed subjects to the study and showed them a 2.5 minute YouTube clip of the first debate to give them an idea of what they should expect to see. Approximately 30 seconds into the clip the experimenter mentioned the “worm” in passing, pointing out how it moved up and down in response to what the leaders said. Our aim here was to show an example of the worm for the benefit of those viewers who had not seen its use in previous debates, and who might otherwise have good reason to suspect that this feature of the broadcast was engineered by us. At this point, the experimenter simply remarked that one of the questions after the debate would be about the worm. Next the experimenter reminded participants the experiment was about memory and that their memory would be tested. As an example, participants were asked to indicate which candidate had used a particular phrase in the clip a few seconds earlier. The participants were told there would be similar questions at the end of the debate and they should pay close attention throughout.

The live coverage was switched on moments after the debate began, allowing us to ensure our worm was in place as soon as the screen became visible. Participants remained in their seats for the duration of the debate, after which the display was turned off and the answer sheets for the “after” questions were distributed. Questions were presented one at a time via a Powerpoint presentation, and were read aloud by a research assistant who was naive as to the manipulation. Answer sheets were then collected and participants were handed response sheets for two final questions which asked if they were suspicious about the worm. After the responses to these questions were collected the experimenter provided participants with a verbal debriefing, explaining that the study was not about memory for political debates but about the role of social influence processes on perceptions of the candidates. We informed participants that the worms were manipulated by us and were not based on the views of undecided voters.

### Questionnaires

Our questionnaires were divided into three parts: Pre-Debate, Post-Debate and Final (Manipulation Check) questions. Pre-debate questions began with demographics (age, gender, occupation, educational qualifications), and then asked participants if they had previously voted in an election, whether or not they planned to vote in the upcoming general election, and who was their current preferred Prime Minister. They were also asked if they had viewed any of the prior election debates and if so in which format (live on television, on a website, or a recording), and if they had previously seen the “worm” graph. Finally, participants were asked to rate on a 1–7 scale how well they expected each of the party leaders to perform in the debate.

Post-debate questions began by asking who won the debate (Putting aside your own party preferences, which party leader do you think “won” this debate? {Brown, Cameron, Clegg, don't know}). Participants were then asked to rate on a 1–5 scale how well each party leader performed relative to their expectations, which of the leaders' policies on the economy appealed to them the most, and which of the three leaders came across as most sincere and trustworthy. Participants were also asked how much the debate would influence their vote on a 1–7 scale and which candidate was their preferred Prime Minister now they had seen the debate. They were asked if they had tracked the worm and who in their opinion had performed best according to the worm, and the extent to which they agreed with the worm. After the responses to the post-debate questions had been collected, participants were asked if they suspected that the worm graph might not correspond to the views of undecided voters (that is, that the experimenters may have manipulated the worm). The last question asked if the worm was biased in favour of any of the party leaders.

### Control sample

The day after the debate, we asked 61 students on the Royal Holloway campus who had viewed the debate but who had not taken part in our study to answer a single question. The students were asked to put aside party preferences and in a secret ballot state who they thought won the debate.

## Results

Our deception was successful: in ratings made following the debate, the majority of viewers said that they did not suspect the worm had been manipulated. A minority (13%) said they were sure that the worm had been manipulated, which may reflect a perceived implausibility in either the appearance or degree of bias of our worms, but may also reflect participants' unfamiliarity with the worm, their knowledge that they were taking part in a psychology experiment and the leading nature of the question (“Did you suspect that the worm graph might not correspond to the views of undecided voters (that is, that the experimenters may have manipulated the worm)?”). We present results from the full sample of 150 participants, but an identical pattern is obtained when analysis is restricted to those participants who did not suspect the worm had been manipulated.

**Figure 2:**
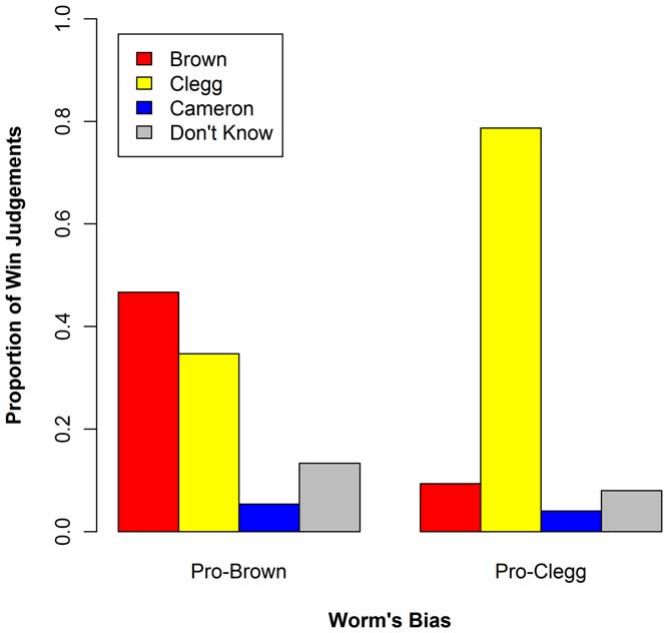
Viewers' perceptions of which candidate won the debate.

The results supported our hypothesis (see Figure 2). In the Brown-biased group, 47% of participants reported that Brown had won the debate (ahead of Clegg on 35% and Cameron on 13%). By contrast, in the Clegg-biased group 79% of participants reported that Clegg had won the debate (ahead of Brown on 9% and Cameron on 4%). Thus, each group selected the winner that was consistent with the bias of the worm that they viewed. The effect of the worm's bias on the judgements of perceived winner was significant, χ^2^(3) = 34.69, *p* = .000, *R* ^2^ = .235 (all *R* ^2^ values we report are Nagelkerke pseudo-*R* ^2^ from multinomial logistic regression analyses; ref. 24). A noteworthy aspect of the data was the relatively poor performance of Cameron, who was widely judged in larger polls to have won the debate [25]–[26]. His poor performance here is consistent with the fact that the worm was biased against him in both groups. However, it could also reflect the characteristics of the present demographic. To test the latter possibility, we polled an independent random sample of 61 Royal Holloway students on the day following the debate. The anonymous responses from this control sample showed a much more even distribution of perceived winners (Figure 2), reinforcing the conclusion that the worm had strong positive and negative influences on judgments of the candidates' performances.

To test the biasing effect of the worm further we constructed a regression model that attempted to predict participants' judgements of the debate winner based on the worm's actual bias, the worm's perceived bias, and participants' prior preference of prime minister. We excluded seven participants who responded ‘Don't Know’ to the question about who won the debate; in the resulting data set, there were 71 participants in the Brown-biased group and 72 in the Clegg-biased group. The full model was statistically significant, χ^2^(14) = 69.01, *p* = .000, *R* ^2^ = 0.456. The predictive power of each individual variable in the model was tested using ratio likelihood tests which computed a chi-square statistic based on the log likelihood difference between the full model and a reduced model that excluded that variable. The results of this analysis are shown in Table 1. As can be seen, the best predictor of participants' judgements of the debate winner was their prior preference of prime minister. For example, of the 48 participants who preferred Clegg prior to the debate, 75% thought that he had won the debate, versus 21% who thought Brown had won. By contrast, of the 25 participants who preferred Brown prior to the debate, 60% thought that he had won the debate, versus 28% who thought Clegg had won. It is not surprising that viewers' opinions of who won the debate are related to those viewers' prior preferences. More importantly, there was a strong, independent effect of the worm's bias. Thus, as can be seen in Table 1, the reduced model which did not include bias (but which did include the other four predictors) accounted for significantly less variance in participants' responses than the full model.

**Table 1:** Likelihood ratio tests of variables in the multinomial regression model predicting debate winner.

Effect	−2 Log Likelihood of Reduced Model	χ^2^	df	*p*
Bias of worm	88.60	10.20	2	.006^**^
Preference before debate	108.37	29.97	6	.000^**^
Perceived worm winner	83.72	5.32	6	.503

The inclusion of the Perceived Worm Winner factor in the full model allowed us to examine the extent to which participants' judgments of who won simply reflected their perception of which candidate was favoured by the worm. Scores on this factor were derived from participants' responses to the post-debate question, “Based on the movements of the worm, which leader do you think did best? {Brown, Cameron, Clegg, don't know}”. The results, shown in Table 1, indicate that the exclusion of this factor from the full model did not significantly affect the amount of variance that could be explained, i.e., participants' explicit perceptions of which candidate was favoured by the worm, did not uniquely predict their judgments of the debate winner. Thus, our manipulation of the worm had effects on people's judgements that went beyond their own reported perceptions of the worm's movements.

We also considered whether the effect of the worm depends on its agreement with the viewer's own judgments. If the viewer perceives the worm to be biased in favour of a specific candidate, she may be more likely to disregard its evaluation. Indeed, such a perception could provoke a reinforcement of the viewer's initial attitudes [27]. To test whether agreement with the worm influenced the results, we considered participants' responses to the following question: “To what extent did you agree with the responses of these undecided voters? (as reflected by the worm)”, rated on a scale from 1 to 7. To facilitate analysis, we collapsed responses into two categories: Low Agreement (1–3 on the original scale) and High Agreement (5–7 on the original scale). We then performed regression analyses separately for these two categories. The Low Agreement condition (*N* = 65) showed the same pattern of results as the full sample, i.e., significant effects of both the worm's bias, χ^2^(2) = 16.36, *p* = .000, and the viewer's prior preference, χ^2^(6) = 24.19, *p* = .000. The High Agreement condition (*N* = 43) showed a different pattern: the worm's bias continued to be a significant predictor, χ^2^(2) = 20.26, *p* = .000, but the viewer's prior preference was not a significant predictor, χ^2^(6) = 24.19, *p* = .619. The latter result is an artefact of the distribution of participants across the two agreement conditions: participants assigned to the High Agreement condition frequently had a prior preference that agreed with the bias of the worm, and hence there was a range restriction issue that reduced the predictive power of the prior preference variable. Critically, though, the bias of the worm significantly affected viewers' judgments even when they claimed to disagree with its evaluations.

It is interesting to consider whether viewers can minimise the influence of the worm by not attending to it. We asked our participants how much they attended to the worm (“Not at all”, “A little”, “Quite often”, or “Attended mostly to the worm”). Relatively few participants (13%) reported attending only a little or not at all to the worm. The worm was extremely salient, often crossing over the heads of the debaters (as in the display used in the ITV broadcast that we modelled our display on), and so it is not surprising that it captured viewers' attention. It remains an open question as to whether a similar influence would be obtained using a more subtle presentation of the worm. However, it is feasible that such a presentation could have an equally strong, or stronger influence. Affective judgments can sometimes be influenced more strongly by subliminal than by supraliminal stimuli [28], and recent research on voting has shown that subtle contextual priming can influence real-world voting [29].

**Figure 3:**
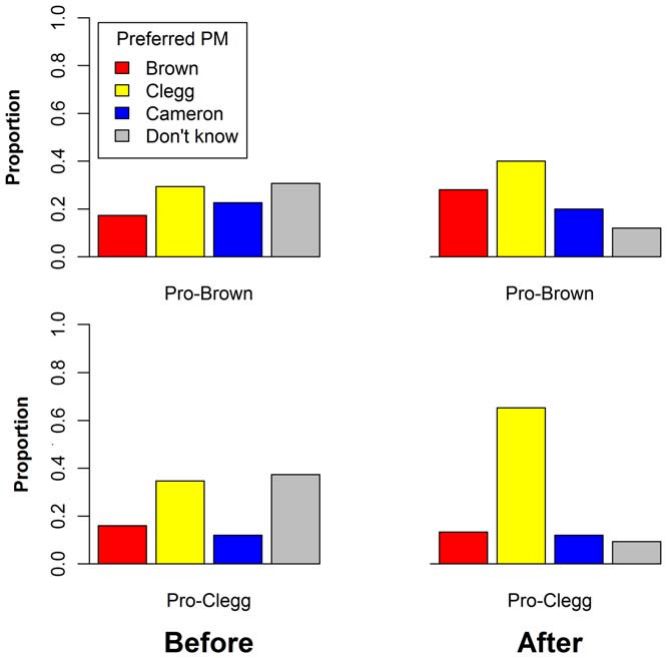
Preferred choice of prime minister for individuals in the two groups, before and after the debate.

The results considered so far show that our manipulation of the worm influenced viewers' judgments of who won the debate. But did the worm also influence viewers' subsequent choices of preferred prime minister? Based on responses given immediately after the debate, the answer is yes. The model that included the worm's bias and the viewer's prior preference as predictors provided a good account of choices of preferred prime minister, χ^2^(12) = 109.00, *R* ^2^ = .569, *p* = .000, and the worm's bias was a significant predictor even after preferred prime minister prior to the debate was partialled out, χ^2^(3) = 10.19, *p* = .017. As can be seen in Figure 3, just over a third of participants were undecided as to their preferred PM prior to the debate, but this proportion decreased to only 10% following the debate. Most of these undecided voters were swayed in the direction of the worm, although there was a general trend for Clegg to become a more popular choice following the debate. However, the effect of the worm remained significant when previously undecided voters were excluded from the analysis, χ^2^(3) = 11.40, *p* = .010. That is, even those individuals who specified prior to the debate a clear preference for which candidate they would like to be prime minister were vulnerable to having their preference modified by the manipulated worm.

It was not possible to determine if the influence of the worm on our participants would persist right through until election day, as there was an ethical requirement to debrief participants following the experiment, ensuring they understood our manipulation of the worm. Prior to debriefing, though, we asked participants, “How much will this debate influence your vote?” They gave ratings between 1 and 7, where 1 indicates “not at all” and 7 indicates “very strongly”. Over half of the sample responded with a rating of 5 or more. When the sample was restricted to those who said that they would vote and had not already submitted a postal vote, 65% gave ratings of 5 or more, and 37% gave ratings of 6 or more. We conclude that watching the debate had a relatively strong influence on voting intentions. This conclusion is consistent with data collected by pollsters following this debate [26], and with previous research [2]–[9].

## Discussion

Our results show that the presence of a worm graph during a televised election debate influences viewers' judgments of who won the debate, who they would prefer to lead the country, and how they intend to vote. The existence of such a social influence effect is consistent with much previous psychological evidence. Nevertheless, it was unclear whether such an effect could occur in the real-world context of an election debate, given the more explicit attempts at influence by the candidates in the debate, the robustness of viewers' political attitudes and the fact that viewers have a personal stake in the election outcome. The surprisingly large effect of our experimental manipulation is therefore of both scientific interest and social importance.

A particularly insidious aspect of the worm's influence is that this influence appears to go beyond the viewer's explicit memory for the worm's movements. That is, the biasing effect of the worm continued to be a significant predictor of judgements of who won the debate even after partialling out the contribution of viewers' perceptions of which candidate was favoured by the worm. This result suggests that the worm's influence may be quite difficult for viewers to discount.

In principle, televised election debates allow voters to form judgements about the leaders and their policies without the filter of (often unbalanced) media sources. Some writers have argued that this absence of “spin” is also a positive aspect of the worm:

I love the crawler and think that it really helps you understand what's going on in the debates – in particular, it helps you take one step back from your own prejudices. It's also just about the only input into debate commentary that comes more or less unmediated; the anonymous “undecided” focus group participants might be dumb or irrational, but they're at least not pushing an agenda. Raw data is always good to have. [30]

According to this perspective, the worm is simply an additional source of “raw data”. Schill and Kirk [10] agree with this perspective, arguing that broadcasting the worm is “fundamentally empowering”, in that “it provides viewers more information to consider when watching the debates and forming their own opinions”. However, we dispute the claim that this is empowering to the viewer. Rather, our results indicate that the presence of the worm makes it more difficult for viewers to form opinions that are truly their own.

Indeed, it is not clear that the worm is a good source of “raw data”. The sample sizes used to produce worm graphs compare unfavourably with sample sizes in the hundreds or thousands that are standard in political polls (which are themselves known to be associated with a considerable degree of error). In the 2010 UK election debates, ITV's worm was based on a sample of only 20 undecided voters and the BBC's worm was based on only 12 undecided voters for each candidate, and as noted above, in the 2008 US Presidential debates CNN used a sample size of 30 voters [31]. Small sample sizes give rise to the genuine possibility that the worm will, by chance, be biased in favour of one of the candidates.

Furthermore, there is also the possibility of systematic sources of bias in the worm. There is generally very little information provided regarding the manner in which undecided voters were selected to generate the worm. When participants are not paid for their time [10], or are paid only a small fee, it is likely that most of those who take part will live in the close vicinity of the broadcasting venue, which may not give rise to a representative sample of the voting population at large. Even with careful sampling, there may be cause to be uncertain of the representativeness of voters who report that they are undecided and yet are sufficiently engaged in the political process to sacrifice their time to participate in debate broadcasts.

Perhaps most worryingly, the technology could be used to systematically distort the outcome of elections. In the United Kingdom, it is not unusual for media organisations to have a specific political alignment; for example, each of the major daily newspapers advised their readers of which of the leaders in the debate would make the best prime minister. Famously, Britain's biggest selling newspaper, *The Sun* (owned by Rupert Murdoch) took credit for having swung the outcome of the closely fought 1992 election [32]. It would seem prudent to avoid a situation in which a media organisation could be accused of having (even inadvertently) manipulated viewers' real-time opinions of televised election debates.

In sum, our data indicate that viewers exposed to the worm are subject to social influence processes which later form the basis of their opinions. Thus, the responses of a small group of individuals could, via the worm, influence millions of voters. This possibility is not conducive to a healthy democracy, and therefore we argue that broadcasters should avoid the simultaneous presentation of average response data with televised election debates.
